# Evaluation of the Anticancer Activity of Calcium Ions Introduced into Human Breast Adenocarcinoma Cells MCF-7/WT and MCF-7/DOX by Electroporation

**DOI:** 10.3390/ph16060809

**Published:** 2023-05-30

**Authors:** Katarzyna Bieżuńska-Kusiak, Julita Kulbacka, Anna Choromańska, Nina Rembiałkowska, Olga Michel, Jolanta Saczko

**Affiliations:** 1 Department of Molecular and Cellular Biology, Wroclaw Medical University, Borowska 211A, 50-556 Wroclaw, Poland; julita.kulbacka@umw.edu.pl (J.K.); anna.choromanska@umw.edu.pl (A.C.); nina.rembialkowska@umw.edu.pl (N.R.); 2 Department of Immunology, State Research Institute Centre for Innovative Medicine, Santariškių 5, 08410 Vilnius, Lithuania; 3 Department of Cytobiochemistry, University of Wroclaw, F. Joliot-Curie 14a, 50-383 Wroclaw, Poland; olga.michel@uwr.edu.pl

**Keywords:** calcium ions, electroporation, breast cancer

## Abstract

Breast cancer ranks among the top three most common malignant neoplasms in Poland. The use of calcium ion-assisted electroporation is an alternative approach to the classic treatment of this disease. The studies conducted in recent years confirm the effectiveness of electroporation with calcium ions. Electroporation is a method that uses short electrical pulses to create transitional pores in the cell membrane to allow the penetration of certain drugs. The aim of this study was to investigate the antitumor effects of electroporation alone and calcium ion-assisted electroporation on human mammary adenocarcinoma cells that are sensitive (MCF-7/WT) and resistant to doxorubicin (MCF-7/DOX). The cell viability was assessed using independent tests: MTT and SRB. The type of cell death after the applied therapy was determined by TUNEL and flow cytometry (FACS) methods. The expression of Cav3.1 and Cav3.2 proteins of T-type voltage-gated calcium channels was assessed by immunocytochemistry, and changes in the morphology of CaEP-treated cells were visualized using a holotomographic microscope. The obtained results confirmed the effectiveness of the investigated therapeutic method. The results of the work constitute a good basis for planning research at the in vivo level and in the future to develop a more effective and safer method of breast cancer treatment for patients.

## 1. Introduction

One of the most common cancers in women, after lung cancer, is breast cancer, which is characterized by a high incidence in all countries [1]. Breast cancer originates in the epithelial cells of the breast gland. It develops locally in the breast and may also metastasize to the lymph nodes and internal organs [2]. Breast cancer accounts for approximately 1.7 million new cases a year, or 25% of all cancers. It is the second-most common cancer in the world [3]. The incidence of breast cancer ranges from 19.4 per 100,000 people in East Africa to 89.7 per 100,000 in Western Europe [4,5]. Currently, the therapy is based on surgical treatment, radiotherapy, and chemotherapy with the use of taxon derivatives. Unfortunately, nearly 70% of patients relapse without responding to standard treatment.

Due to the constantly growing number of new cases and the low effectiveness of traditional therapy, there is a need to search for less toxic and more effective solutions. Electrochemotherapy (ECT) is a relatively new anticancer treatment. Short electric pulses can induce temporary permeabilization of the cell membranes. This phenomenon, called electroporation (EP), results in the increased transport of molecules into the tumor cells. It was noted that electropermeabilization could firmly enhance the efficacy of various drugs such as catechin [6], cisplatin [7,8,9], doxorubicin [10,11], or calcium ions [12,13].

The calcium ion (Ca^2+^) concentration is higher in the extracellular space than in the cell’s interior. This cellular Ca^2+^ homeostasis is tightly regulated by ATP (adenosine triphosphate)-dependent calcium pumps, Ca^2+^-ATPases. Calcium ions perform various functions in the human body. They are involved in many cellular processes, such as cell cycle regulation, apoptosis, and proliferation. Calcium ion signaling requires close cooperation between cells and organelles; it is, in fact, a very sophisticated mode of communication to maintain whole cell homeostasis and functionality. Much attention has been paid to the collaboration between the ER (endoplasmic reticulum) and mitochondria, which interact through highly dynamic Ca^2+^ transport systems [14,15,16,17]. Signal transduction takes place through channels in the ER membrane that are sensitive to local Ca^2+^ increases [18]. After many years of intensive research into the role of calcium ions in the stress response and cell death, it has become clear that the changes disrupting cell homeostasis are largely due to abnormal calcium signaling. Depending on the degree of disturbance of cell homeostasis, it can be regulated by repair mechanisms or induce the path of cell death (depending on the intensity of damage, the most common path is apoptotic or necrotic) [19]. The ability to regulate intracellular pathways such as proliferation and apoptosis suggests that calcium ion modulation in neoplastic cells could be used as a therapeutic option [20].

Calcium electroporation (CaEP) is an emerging technique used in cancer therapy that combines electroporation with the administration of calcium ions (Ca^2+^). It has shown promising results in the treatment of various types of tumors, including melanoma, breast cancer, and sarcoma [12,13,20]. The mechanism of action of CaEP involves several steps that can distinguish it from other electroporation-based therapies. Firstly, CaEP, or electroporation, is the initial step that facilitates the entry of calcium ions into the cancer cells. This phenomenon causes rapid and increased calcium ion influx through temporary pores. It is commonly known that calcium plays a crucial role in multiple cellular processes and signaling pathways. The sudden influx of calcium ions into the cancer cells significantly disrupts intracellular calcium homeostasis [18]. Cells normally maintain low cytosolic calcium concentrations compared to the extracellular environment. The elevated intracellular calcium levels trigger a series of signaling cascades and cellular responses, including disruption of mitochondrial function, leading to mitochondrial membrane depolarization and the release of pro-apoptotic factors, initiating the programmed cell death pathway [12,19]. Additionally, CaEP affects calcium release from the ER, causing ER stress and the unfolded protein response (UPR). These signals can lead to cell cycle arrest, apoptosis, or autophagy [17]. Moreover, calcium ion overload can induce the generation of reactive oxygen species (ROS) within the cells, resulting in oxidative stress [14]. One of the latest phenomena observed post-CaEP is immunogenic cell death (ICD), which is a form of cell death that elicits an immune response against tumor cells. It was noted that CaEP-induced cell death can release danger-associated molecular patterns (DAMPs) and activate the immune system, promoting the recognition and elimination of tumor cells by immune cells [21]. The other important issue is that calcium channels, including alpha1G (Cav3.1) and alpha1H (Cav3.2), play a critical role in cellular signaling and homeostasis. Studies have revealed that the expression and activity of alpha1G and alpha1H channels are altered in breast tumors, suggesting their potential involvement in cancer progression [1,5]. In the context of calcium electroporation for cancer therapy, these T-type calcium channels have emerged as important targets. Notably, the use of calcium channel blockers, which specifically inhibit the activity of these channels, holds promise for enhancing the efficacy of calcium electroporation by further sensitizing cancer cells to calcium-induced cytotoxicity [18].

Thus, CaEP, in relation to other electroporation-based therapies, is a specific method that triggers specific cellular responses that can enhance the effectiveness of cancer treatment. The disruption of calcium homeostasis and the subsequent activation of various signaling pathways differentiate CaEP from other electroporation-based therapies, providing a unique mechanism of action for targeted cancer treatment.

The aim of the study was to evaluate the antitumor potential of electroporation in combination with calcium ions in relation to sensitive human mammary gland adenocarcinoma (MCF-7/WT) and doxorubicin-resistant (MCF-7/DOX) cell lines.

## 2. Results

### 2.1. MTT Assay

The MTT (3-(4,5-dimethylthiazol-2-yl)-2,5-diphenyltetrazolium bromide) assay assessed the viability of MCF-7/WT and MCF-7/DOX after all experiments. The obtained results were expressed as the percentage of viable cells relative to untreated control cells.

**Figure 1:**
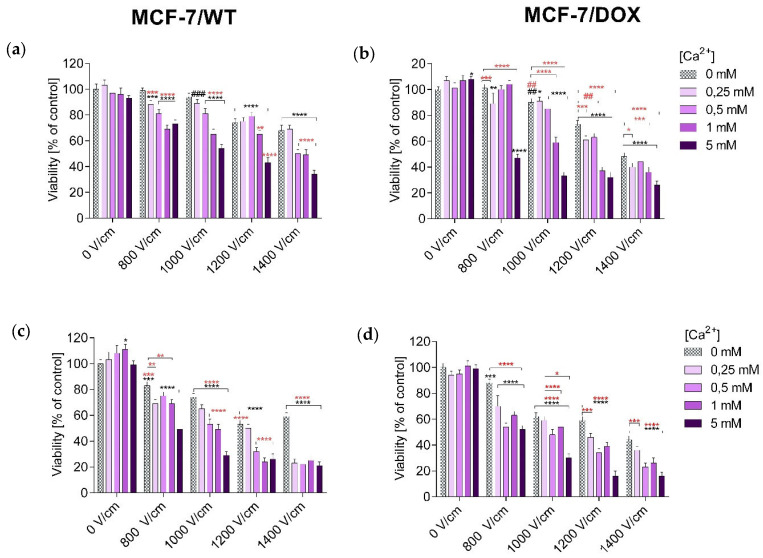
Survival of MCF-7/WT and MCF-7/DOX cells determined by the MTT test after 24-h (**a**,**b**) and 48-h (**c**,**d**) incubation, depending on the concentration of Ca^2+^ ions in combination with electroporation. Survival expressed as a percentage of control (untreated) cells. Error bars are means ± SD for n = 3. Statistical significance for the group of control cells was determined by the Sidak test, where: *** statistically significant for *p* = 0.0002, **** statistically significant for *p* < 0.0001, ### statistically significant for *p* = 0.0006. Statistical significance for the group of cells subjected to electroporation was determined by the Sidak test, where: ** statistically significant for *p* = 0.006, *** statistically significant for *p* = 0.0006, **** statistically significant for *p* < 0.0001 (MCF-7/WT, (**a**)). *** Statistically significant for *p* = 0.0009, **** statistically significant for *p* < 0.0001. Statistical significance for the group of cells subjected to electroporation was determined by the Sidak test, where: ** statistically significant for *p* = 0.0083, *** statistically significant for *p* = 0.0009, **** statistically significant for *p* < 0.0001 (MCF-7/WT, (**c**)); * statistically significant for *p* = 0.0151, ** statistically significant for *p* = 0.0019, **** statistically significant for *p* < 0.0001, # statistically significant for *p* = 0.0182, ## statistically significant for *p* = 0.0055. Statistical significance for the group of cells subjected to electroporation was determined by the Sidak test, where: * statistically significant for *p* = 0.0385, *** statistically significant for *p* = 0.0006, **** statistically significant for *p* < 0.0001, ## statistically significant for *p* = 0.0055 (MCF-7/DOX, (**b**)); *** statistically significant for *p* = 0.0004, **** statistically significant for *p* < 0.0001. Statistical significance for the group of cells subjected to electroporation was determined by the Sidak test, where: * statistically significant for *p* = 0.0286, *** statistically significant for *p* = 0.0001, **** statistically significant for *p* < 0.0001 (MCF-7/DOX, (**d**)).

The number of viable cells in both tested cell lines was determined after 24 and 48 h of incubation, respectively. Values showing the numbers of viable cells after EP are plotted in the columns labeled “0 mM” in the graphs showing the relationship of cell survival to CaEP. Values showing the numbers of viable cells after incubation in CaCl_2_ are plotted in the columns labeled “0 V/cm” in the graphs. Incubation of MCF-7/WT cells in calcium chloride solutions (0.25 mM; 0.5 mM; 1 mM; 5 mM) did not cause a significant decrease in survival. After 24 h of incubation, cell survival was, respectively, 103 ± 4; 97 ± 5; 96 ± 5; and 93 ± 2 (Figure 1a). The 48-h post-therapy had a stimulating effect on the cells, increasing their proliferation: 103 ± 6; 108 ± 6; 111 ± 4; 99 ± 3 (Figure 1c). The incubation in calcium chloride solutions did not have any significant cytotoxic effect; on the contrary, it caused increased cell proliferation after 48 h. After 24 h in the case of the MCF-7/DOX cell line, a slight increase in the number of cells was observed: 107 ± 3; 101 ± 4; 107 ± 4; 108 ± 2 (Figure 1b). After 48 h, the values were: 94 ± 3; 95 ± 3; 101 ± 4; 99 ± 3 (Figure 1d).

It is clearly visible that after the application of CaEP, even at the voltages of 800 and 1000 V/cm, a significant decrease in the number of living cells was observed. For the MCF-7/WT cell line, cell survival after 24 h was: 800 V/cm + 1 mM (66 ± 3), 800 V/cm + 5 mM (70 ± 4), 1000 V/cm + 1 mM (62 ± 4), 1000 V/cm + 5 mM (56 ± 3) (Figure 1a). After 48 h, the values were 800 V/cm + 5 mM (49 ± 3), 1000 V/cm + 1 mM (49 ± 4), and 1000 V/cm + 5 mM (29 ± 3), respectively (Figure 1c). For the MCF-7/DOX cell line, there was also a significant decrease in the number of viable cells. Viability after 24 h of incubation was: 800 V/cm + 5 mM (47 ± 3), 1000 V/cm + 5 mM (33 ± 3) (Figure 1b). After 48 h of incubation: 1000 V/cm + 0.5 mM (48 ± 4), 1000 V/cm + 5 mM (30 ± 3) (Figure 1d).

For the MCF-7/WT cell line, the use of EP increased the cytotoxicity compared to calcium chloride. The lowest number of viable cells was recorded for values of 1200 and 1400 V/cm, when the number of viable cells dropped to 53% and 59%, respectively. For the MCF-7/DOX cell line, after applying EP, a decrease in the number of living cells was also observed, along with an increase in the parameters of the electric field strength. The smallest number of living cells was recorded at an electric field strength of 1400 V/cm- 44%.

The highest decrease in cells’ mitochondrial activity occurred after the use of CaEP. Based on the results presented above, in the MCF-7/WT cell line, the greatest decrease in the number of viable cells took place after the following electroporation parameters were used: 1200 V/cm + 5 mM, 1400 V/cm + 1 mM, and 1400 V/cm + 5 mM after 24-hour incubation. A 48-h incubation caused a further increase in cytotoxicity, which was observed for all electroporation parameters during incubation in 5 mM solutions and at the highest electroporation parameter. For the 24-h incubation, the number of viable cells dropped to 34% and, after 48 h, to 21% (Figure 1a,b).

For the MCF-7/DOX cells, there was a significant decrease in the number of viable cells after incubation for 24 h with CaEP (EP parameters during incubation in 5 mM solutions and with 1400 V/cm). After 48 h of incubation, the survival decreased even more, so that at 1400 V/cm + 5 mM, the survival dropped to 16% (Figure 1c,d).

The results suggest that cells of the MCF-7/DOX line are more sensitive to CaEP.

### 2.2. SRB Assay

The SRB (sulforhodamine B) assay assessed the viability of MCF-7/WT and MCF-7/DOX after EP, CaEP, and incubation with calcium chloride. The obtained results were expressed as the percentage of viable cells relative to untreated control cells. The SRB test results confirmed the MTT assay results. 

**Figure 2:**
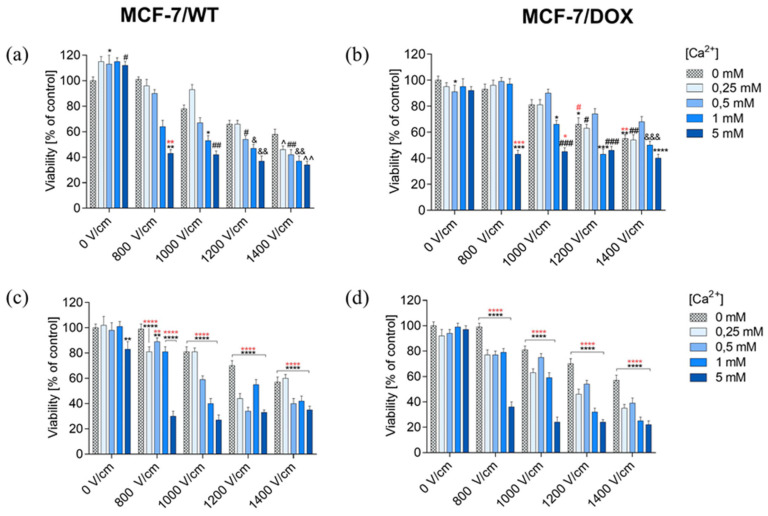
Survival of MCF-7/WT and MCF-7/DOX cells determined by the SRB test after 24-h (**a**,**b**) and 48-h (**c**,**d**) incubation depending on the concentration of Ca^2+^ ions in combination with electroporation. Survival expressed as a percentage of control (untreated) cells. Error bars are means ± SD for n = 3. Statistical significance for the group of control cells was determined by the Sidak test, where: * statistically significant for *p* = 0.0336, ** statistically significant for *p* = 0.0062, # statistically significant for *p* = 0.0393, ## statistically significant for *p* = 0.0052, & statistically significant for *p* = 0, 0125, && statistically significant for *p* = 0.0021, ^ statistically significant for *p* = 0.0105, ^^ statistically significant for *p* = 0.0012. Statistical significance for the group of cells subjected to electroporation was determined by the Sidak test, where: ** statistically significant for *p* = 0.0052 (MCF-7/WT, (**a**)). ** statistically significant for *p* = 0.0021, **** statistically significant for *p* < 0.0001. Statistical significance for the group of cells subjected to electroporation was determined by the Sidak test, where: ** statistically significant for *p* = 0.0058, **** statistically significant for *p* < 0.0001 (MCF-7/WT, (**c**)). * Statistically significant for *p* = 0.0355, ** statistically significant for *p* = 0.0026, *** statistically significant for *p* = 0.0001, **** statistically significant for *p* < 0.0001. # statistically significant for *p* = 0.0182, ## statistically significant for *p* = 0.002, ### statistically significant for *p* = 0.0002, &&& statistically significant for *p* = 0.0007. Statistical significance for the group of cells subjected to electroporation was determined by the Sidak test, where: * statistically significant for *p* = 0.0228, ** statistically significant for *p* = 0.0026. *** statistically significant for *p* = 0.0007, # statistically significant for *p* = 0.0355 (MCF-7/DOX, (**b**)). **** statistically significant for *p* < 0.0001. Statistical significance for the group of cells subjected to electroporation was determined by the Sidak test, where: **** statistically significant for *p* < 0.0001 (MCF-7/DOX, (**d**)).

Values showing the number of viable cells after applying electroporation (800 V/cm, 1000 V/cm, 1200 V/cm, 1400 V/cm, 8 pulses, 100 µs pulse duration) are presented in the graphs in the columns labeled “0 mM” in graphs showing the dependence of cell survival on CaEP. Values showing the numbers of viable cells after incubation in CaCl_2_ are plotted in the columns labeled “0 V/cm” in the graphs. Incubation in CaCl_2_ solutions did not reduce the viability of the MCF-7/WT cell line. After 24 h of incubation, cell survival was, respectively: 103 ± 4; 97 ± 5; 96 ± 5; 93 ± 2 (Figure 2a). The 48-h incubation had a stimulating effect on the cells, increasing their proliferation. Survival values were as follows: 103 ± 6; 108 ± 6; 111 ± 4; 99 ± 3 (Figure 2c). Incubation of MCF-7/DOX cells in calcium chloride solutions did not result in any significant changes in the number of viable cells. After 24 h of incubation, only a slight increase in cell number was observed; the values were: 107 ± 3; 101 ± 4; 107 ± 4; 108 ± 2 (Figure 2b). After 48 h, a slight decrease in the number of viable cells was observed. Survival values were as follows: 94 ± 3; 95 ± 3; 101 ± 4; 99 ± 3 (Figure 2d).

Therefore, incubation in calcium chloride solutions did not have any significant cytotoxic effect; after 24 h, a slight increase in cell proliferation was observed. The results for the MCF-7/DOX cell line were similar.

For the MCF-7/WT cell line, the application of electric pulses decreased the survival rate with an increase in electric field strength parameters. After 48 h of incubation, a cell viability decrease was obtained. In the MCF-7/DOX cell line, a decrease in cell viability was also noted after the use of EP, in particular after 48-h incubation. Consequently, the increasing EP intensities resulted in decreasing cell viability in both cell lines. The MCF-7/DOX line turned out to be more sensitive to the applied EP. The lowest survival rate was recorded for the 1400 V/cm electric field intensity.

It is clearly visible that the survival of MCF-7/WT cells significantly decreased after the application of CaEP. Even at lower values of the applied voltage, there is a clear decrease in the number of viable cells. The number of viable cells after 24 h of incubation was: 800 V/cm (43 ± 3), 1000 V/cm + 5 mM (42 ± 3) (Figure 2a). After a 48-h incubation, there was a stronger decrease in the cell’s survival, in particular for the following parameters: 800 V/cm + 5 mM (30 ± 4), 1000 V/cm + 1 mM (40 ± 4), and 1000 V/cm + 5 mM (27 ± 4) (Figure 2c).

For MCF-7/DOX cells, 24 h after the CaEP application, the values were as follows: 800 V/cm + 5 mM (43 ± 3), 1000 V/cm + 5 mM (45 ± 3) (Figure 2b). In the case of a 48-h incubation, the values were: 800 V/cm (36 ± 4), 1000 V/cm + 5 mM (24 ± 4) (Figure 2d). This significant viability drop indicated the anticancer potential of calcium electroporation in breast cancer cells.

Based on the results presented above, in the MCF-7/WT cell line, 24 h after CaEP application, the survival rate decreased below 50% for all electroporation parameters during incubation in 5 mM solutions at 1400 V/cm electric field intensity (Figure 2a). The 48-h incubation resulted in a further increase in the cytotoxicity of CaEP (Figure 2c). For 24-h incubation with the highest EP parameter, the survival rate decreased by 66%, and after 48 h, by as much as 73%. For the MCF-7/DOX cell line, cell survival decreased after 24 h of incubation after CaEP application. The lowest number of viable cells was observed for all electroporation parameters during incubation in 5 mM solutions (Figure 2b). After 48 h of incubation, the survival rate decreased even more, especially after incubation in 5 mM solutions at a voltage of 1400 V/cm (Figure 2d). After 24 h of incubation, cell viability decreased by 60% (with the highest EP parameter), and after 48 h by even 78% (1400 V/cm + 5 mM).

The results suggest that the MCF-7/DOX line is more sensitive to CaEP, especially at the highest electroporation parameter, 1400 V/cm.

### 2.3. TUNEL Assay

**Figure 3:**
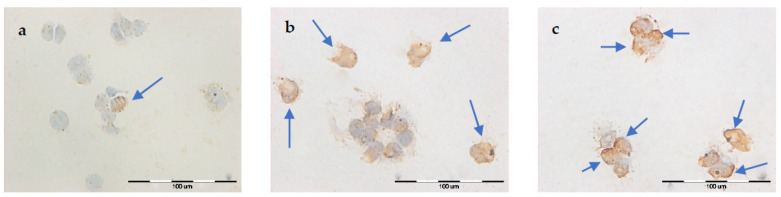
Evaluation of apoptosis by TUNEL method in MCF-7/WT cell line after EP: (**a**) 1000 V/cm, (**b**) 1200 V/cm, (**c**) 1400 V/cm.

**Figure 4:**
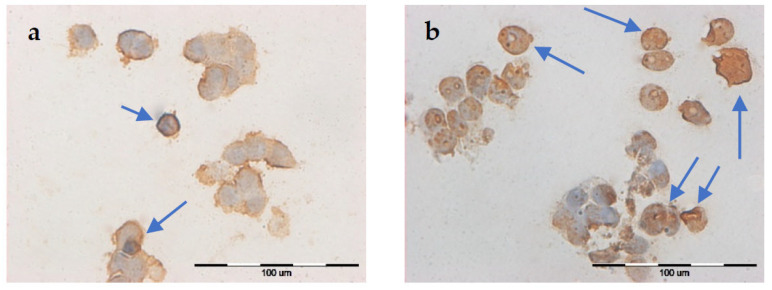
Evaluation of apoptosis using the TUNEL method in MCF-7/DOX cell line after EP: (**a**) 1000 V/cm, (**b**) 1400 V/cm.

**Figure 5:**
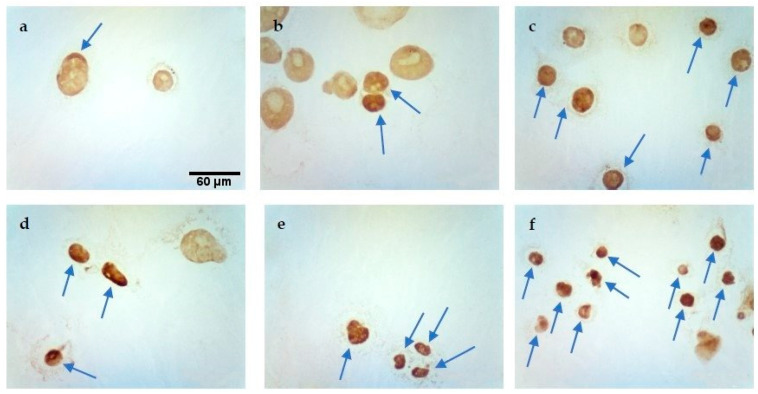
Evaluation of apoptosis by TUNEL method in MCF-7/WT cell line after CaEP: (**a**) 800 V/cm + 5 mM, (**b**) 1000 V/cm + 1 mM, (**c**) 1200 V/cm + 0.5 mM, (**d**) 1200 V/cm + 5 mM, (**e**) 1400 V/cm + 0.5 mM, (**f**) 1400 V/cm + 5 mM. Scale bars correspond to 60 µm.

**Figure 6:**
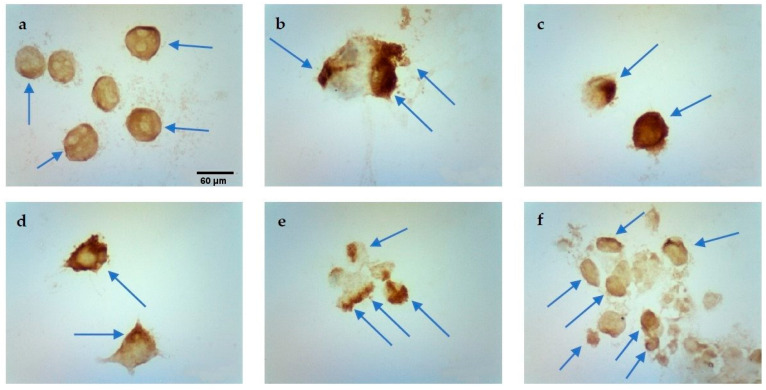
Evaluation of apoptosis using the TUNEL method in MCF-7/DOX cell line after CaEP: (**a**) 1000 V/cm + 1 mM, (**b**) 1200 V/cm + 0.5 mM, (**c**) 1200 V/cm + 1 mM, (**d**) 1200 V/cm + 5 mM, (**e**) 1400 V/cm + 0.25 mM, (**f**) 1400 V/cm + 5 mM. Scale bars correspond to 60 µm.

The percentage of positively stained nuclei was analyzed (cells marked with an arrow). Incubation in calcium chloride solutions did not induce the apoptosis process. On the other hand, both EP and CaEP induced cell apoptosis, with a small percentage of stained nuclei after EP (Table 1). The number of apoptotic cells increased with the intensity of the electric field during EP. An increased number of positively stained nuclei was noted after the application of CaEP compared to EP alone (Figure 3, Figure 4, Figure 5 and Figure 6). The MCF-7/DOX cell line had a lower percentage of apoptotic cells than the MCF-7/WT line.

### 2.4. Assessment of the Type of Induced Cell Death and the Percentage of Individual Cells after CaEP Application (FACS Method)

**Figure 7:**
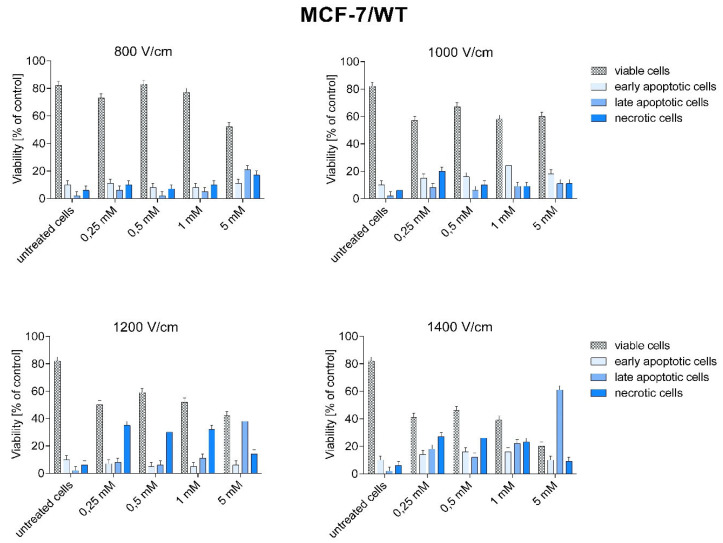
Types of cell death after CaEP application in MCF-7/WT cell line. A number expressed as a percentage of cells: early apoptotic (Sytox −, Annexin V +), living (Sytox −, Annexin V −), late apoptotic (SYTOX +, Annexin +), necrotic (Sytox +, Annexin −).

**Figure 8:**
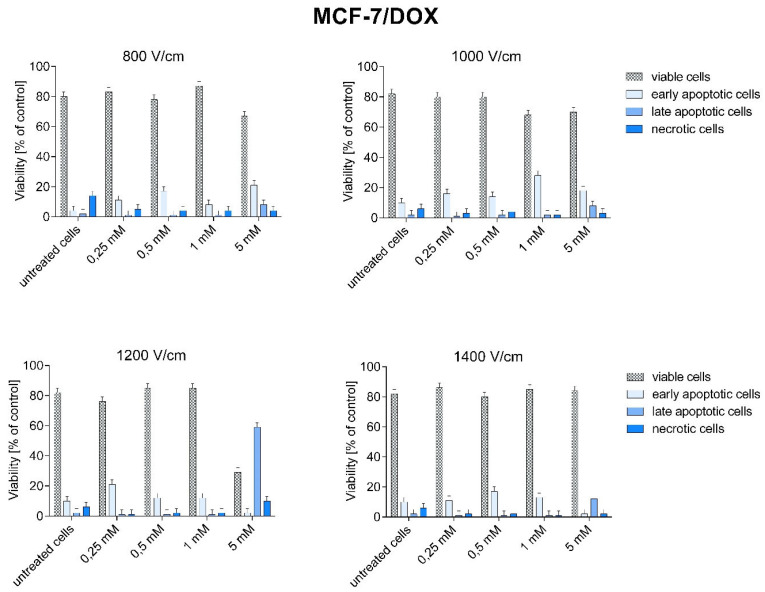
Types of cell death after CaEP application in MCF-7/DOX cell line. Number expressed as a percentage of cells: early apoptotic (Sytox −, Annexin V +), living (Sytox −, Annexin V −), late apoptotic (SYTOX +, Annexin +), necrotic (Sytox +, Annexin −).

After CaEP, cells of both lines were subjected to cytometric analysis of double staining: annexin V-APC and SYTOX*^TM^* Green. Figure 7 and Figure 8 show the percentage of live, necrotic, and apoptotic cells.

In MCF-7/WT cells, late-apoptotic and necrotic cell growth was observed after CaEP application. The increase in the number of late apoptotic cells in the MCF-7/WT cell line at 1200 and 1400 V/cm is most likely related to the increased electric field strength.

The highest percentage of late apoptotic cells was obtained for 1400 V/cm + 5 mM (61.2%), 1200 V/cm + 5 mM (38.2%), 1400 V/cm + 1 mM (22%), and 800 V/cm + 5 mM (21.2%). The number of necrotic cells was high after using the following parameters: 1200 V/cm and 1400 V/cm (from 23.4% to 35%) (Figure 7). The number of living cells decreased with increasing electric field strength. The smallest number of living cells was observed for the parameter 1400 V/cm.

In the MCF-7/DOX cell line, the number of dead cells was lower than in the MCF-7/WT line. The highest percentage of dead cells were cells in early apoptosis (except for the parameter 1200 V/cm + 5 mM). The number of late apoptotic and necrotic cells was lower than the early apoptotic ones. The electric field strength values did not affect the number of apoptotic cells. A high percentage of early apoptotic cells was demonstrated for the following CaEP parameters: 800 V/cm + 5 mM (20.9%), 1000 V/cm + 1 mM (27.9%), 1200 V/cm + 0.25 mM (21.2%), and 1400 V/cm + 0.5 mM (17.5%). The lowest number of viable cells was noted for the parameter 1200 V/cm + 5 mM (29.4%) (Figure 8).

As can be seen, the data on the number of apoptotic cells in the MCF-7/DOX line is lower compared to the other cell line, which is most likely related to their resistance to doxorubicin.

### 2.5. Immunocytochemical Evaluation of Cav3.1 Subunit Expression of T-Type Calcium Channel

In Table 2, the intensity of the immunocytochemical reaction and the percentage of cells stained positively in both cell lines are presented. Immunocytochemical staining studies revealed the expression of Cav3.1, a T-type calcium channel protein, in the MCF-7/WT cell line (Figures S1 and S2) and MCF-7/DOX (Figures S3 and S4) after EP and CaEP application.

No immunocytochemical reaction was observed in MCF-7/WT cells incubated in CaCl_2_ solutions. After EP alone was applied, a significant increase in the immunocytochemical reaction intensity was observed. The number of positively stained cells rose with the intensity of the electric field. CaEP induces an increase in the expression of the Cav3.1 protein, and the intensity of the reaction also rises with the increase in the electric field. The intensity of the reaction is very strong in cells after CaEP in 1 mM and 5 mM CaCl_2_ solutions. The percentage of positively stained cells ranged from 90 to 100 (Table 2). After 48 h of incubation, the expression of the Cav3.1 subunit increased very slightly.

In the MCF-7/DOX cell line, the percentage of positively stained cells is slightly higher for most parameters than in the MCF-7/WT line. Cells incubated in calcium chloride solutions showed no reaction. The use of EP resulted in a marked increase in the expression of the Cav3.1 subunit. The application of CaEP increased the expression of the Cav3.1 protein. The intensity of the reaction and the percentage of positively stained cells also increase with the intensity of the electric field. The highest expression of the Cav3.1 subunit of the T-type calcium channel was observed in the MCF-7/DOX cell line after 48 h of incubation.

### 2.6. Immunocytochemical Evaluation of Cav3.2 Subunit Expression of T-Type Calcium Channel

In Table 3, the intensity of the immunocytochemical reaction and the percentage of cells stained positively in both cell lines are presented. Figures S5 and S6 show the expression of the Cav3.2 T-type calcium channel protein in the MCF-7/WT cell line, while Figures S7 and S8 show the expression of the same protein in MCF-7/DOX cells.

In the MCF-7/WT cell line, the percentage of positively stained cells increased during the longer incubation time. For most parameters, the reaction was slightly weaker compared to the assays with the analyzed expression of the Cav3.1 subunit. The percentage of cells stained positively was similar to that of the Cav3.1 subunit. A very strong positive reaction was observed after CaEP in 0.5 mM, 1 mM, and 5 mM calcium chloride solutions. The percentage of positively stained cells was very high, from 90 to 100% (Table 3). After 48 h of incubation, the intensity of the reaction increased.

In the MCF-7/DOX cell line, the percentage of positively stained cells was higher for most parameters compared to the MCF-7/WT line. The EP application increased the expression of the Cav3.2 subunit. For a 48-h incubation time, a positive immunocytochemical reaction was observed only for the parameters 1200 and 1400 V/cm. The percentage of positively stained cells increased with increasing electric field intensity. CaEP causes a strong expression of the Cav3.2 protein. The intensity of the reaction and the number of positively stained cells also increased with the rise in the electric field strength (Table 3). The higher the EP parameter used, the more intense the immunocytochemical reaction and the greater the percentage of positively stained cells observed.

MCF-7/DOX cells showed higher expression of the Cav 3.2 subunit of the T-type calcium channel compared to MCF-7/WT cells.

### 2.7. Digital Holographic Microscopy (DGM) Study

A digital holographic microscopy (DGM) study showed morphological changes in MCF-7/WT and MCF-7/DOX cell lines. The experiments included two groups of cells: after EP and after CaEP. Controls incorporated untreated cells. After the therapeutic method was applied, the cells were incubated for 24 h prior to analysis.

**Figure 9:**
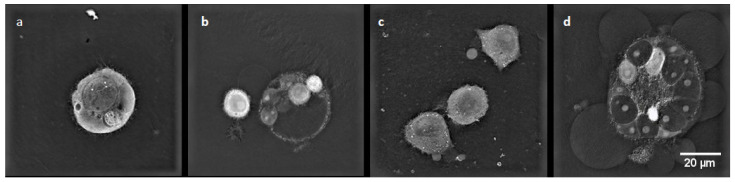
The morphology of the MCF-7/WT cells: (**a**) control cells; (**b**) cells after EP 1200 V/cm; (**c**) cells after CaEP 1200 V/cm + 1 mM; (**d**) cells after CaEP 1400 V/cm + 5 mM (×60).

MCF-7/WT control cells (Figure 9a) were in good condition and had a normal morphological structure: the cell nucleus had a regular shape, and mitochondria and vacuoles were visible in the cytoplasm. After electroporation (Figure 9b), fibrous elements within the cytoplasm were observed in the cells. The most remarkable changes in cell structure were noticeable after CaEP when an outflow of cytoplasm in the form of many obstructed vesicles was observed (Figure 9c,d). Such changes are very characteristic of the phenomenon of apoptosis. Figure 9 shows a group of cells forming apoptotic bodies. Clusters of small lysosomal vesicles located on the inside of the cell membrane were also observed.

**Figure 10:**
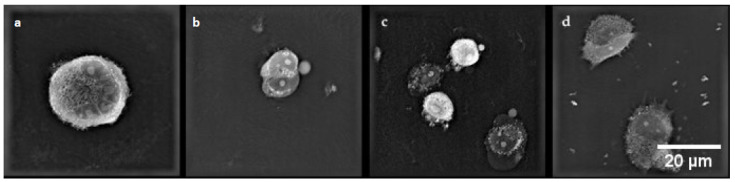
Cell morphology of the MCF-7/DOX cell line. (**a**) control cells; (**b**) cells after EP 1400 V/cm; (**c**) cells after CaEP 1200 V/cm + 5 mM; (**d**) cells after CaEP 1200 V/cm + 5 mM (×60).

In the control cells of the MCF-7/DOX cell line (Figure 10a), cell nuclei of irregular shape and small vacuoles were visible in the cytoplasm. After electroporation (Figure 10b), characteristic apoptotic bodies were formed. After applying CaEP (Figure 10c,d), the cells lost their cell membrane integrity, resulting in the condensation of fibrous material in the cytoplasm and the formation of apoptotic bodies.

## 3. Discussion

Breast cancer is the most common malignant tumor in women in the world and the second most common cause of cancer death [2,5,22]. The prognosis depends on the tumor stage. The main problem in treating breast cancer is the late detection of the tumor, which increases the probability of metastasis [23]. Calcium electroporation (CaEP) can be a new possibility for more effective and safer treatment of breast cancer. Calcium ions in calcium chloride solution, which under normal conditions poorly penetrate cell membranes, were selected for the experiments. These ions were introduced into the cells by electroporation (EP). The differences in the response of two cell lines were determined: sensitive (MCF-7/WT) and resistant to doxorubicin (MCF-7/DOX). The concentration of CaCl_2_ solutions and the EP parameters used in this work were selected based on the results of other research teams.

Convergent results of the MTT and SRB tests were obtained. Both adenocarcinoma cell lines turned out to be sensitive to electroporation and more sensitive to calcium electroporation. Incubation in calcium chloride solutions did not cause a cytotoxic effect. Similar results were obtained by Romero S. et al., who investigated the effect of EP with calcium ions on A431 (human epidermoid carcinoma) and HL-60 (human leukemia) cells. In both cell lines, calcium ions had no significant effect on cell viability at all concentrations tested (1, 3, 5, and 10 mmol /L), as evaluated after 24 h of incubation [24]. Calcium concentration is in the balance between the external and internal environments of the cell. Therefore, under physiological conditions, its transport to the cell is difficult. Calcium ions cannot affect the cells alone. Treating cells with electrical impulses alone caused a reduction in their metabolic activity. The most significant decrease in survival occurred at 1200 and 1400 V/cm in both cell lines. The results of the SRB test were similar to those obtained with the MTT assay. The results of the MTT test after the application of CaEP showed that for the MCF-7/WT cell line after 24 h of incubation, the least viable cells were observed after using the following electroporation parameters and 5 mM calcium concentration: 1200 V/cm + 5 mM, 1400 V/cm + 1 mM, and 1400 V/cm + 5 mM. The longer 48-h incubation resulted in a further reduction of the metabolic activity of the cells. The analysis with the SRB test showed that the highest decrease in survival occurred after all parameters of CaEP at a concentration of 5 mM and 1400 V/cm. After 24 h of incubation with the highest EP parameter, the survival rate decreased to 34% and, after 48 h, to 35%. In the case of the MCF-7/DOX cell line, the number of viable cells was reduced even to 26% at the highest electroporation parameter. After 48 h of incubation, survival decreased even more. At 1400 V/cm + 5 mM, the number of viable cells was 16%. The SRB test, performed after 24-h incubation, showed a significant reduction in survival for all parameters of CaEP at a concentration of 5 mM (the most significant decrease was recorded for the parameter 1400 V/cm + 5 mM, where the survival rate was 40%). After 48 h of incubation, cell survival was even lower. After applying 1400 V/cm + 5 mM, the viability was 22%.

Gehl et al. found that treating cells with calcium chloride did not affect the survival of cells in the MDA-MB231 cell line (human breast adenocarcinoma cells), while CaEP caused a sharp decrease in survival. After performing the MTT test for cells subjected to CaEP, where CaCl_2_ was used at a concentration of 0.5 mM, the survival decreased to 42%. For 1 mM and 5 mM solutions, the number of viable cells dropped below 40% [25]. The same research team compared calcium electroporation with electrochemotherapy in studies using three cancer cell lines (DC-3F-hamster lung cancer cell line, K-562-human leukemia line, and mouse Lewis lung cancer cell line). Studies have shown that CaEP significantly reduces the survival of cancer cells. Even CaEP at a 0.5 mM concentration of calcium ions markedly decreased cell survival [26].

The results of in vitro studies in cell monolayers show that, unlike cisplatin and bleomycin, electroporation with calcium ions induces cell death without causing genotoxicity. The cytotoxicity of this method correlates with a sudden, large decrease in the mitochondrial membrane potential and depletion of the ATP pool [27]. The complete understanding of the molecular mechanisms responsible for the important mechanism of Ca^2+^ influx was not completed until 2006 [28]. Voltage-gated calcium channels (T-VGCC) are encoded by genes in Cav3 subunits [29]. Altered expression of specific calcium-permeable ion channels is seen in some breast tumors [30]. There are few studies on the effect of CaEP on the expression of Cav3.2 subunits of T-type calcium channels. Taylor et al. investigated the relationship between T-type calcium channels and cell proliferation. It turned out that the mRNA level for the Cav3.1 and Cav3.2 calcium channel subunits is high in MCF-7 cells. Interestingly, silencing of the genes determining the expression of T-type calcium channels reduced the proliferation of MCF-7 cells by as much as 45% compared to the control [31]. In similar studies, Ohkubo et al. showed that the voltage-activated Cav3.1 calcium channel is involved in the inhibition of proliferation and apoptosis in human MCF-7 breast cancer cells. Studies conducted in recent years indicate T-type calcium channels as a molecular target in anticancer therapy [32]. The results of other research teams also indicate the possibility of selectively inducing the death of cancer cells by activating the expression of VGCC in human breast cancer cells. Importantly, this mechanism is independent of the breast cancer subtypes [33].

In this paper, the effect of an increased influx of calcium ions into the cell on the expression of T-type calcium channel subunits (Cav3.1 and Cav3.2) was investigated. The results of immunocytochemical tests are presented in Table 3. The electroporation itself increased the expression of the Cav3.2 subunit. Moreover, the use of CaEP led to a substantial increase in the expression of both examined subunits.

In the case of the Cav3.1 subunit, moderately intense staining in 90–95% of cells was noted in the cells of the MCF-7/WT line after the application of 1400 V/cm. In the MCF-7/DOX cell line, electroporation at 1200 V/cm and 1400 V/cm caused a strong positive reaction in 90% of cells. After CaEP, a very high increase in the expression of the Cav3.1 subunit was observed. In the MCF-7/WT cell line, the percentage of stained cells was 98–100% after EP with 1 mM and 5 mM calcium chloride, regardless of the applied voltage. In the MCF-7/DOX cell line, there was also a strong increase in the expression of the Cav3.1 subunit. A significant reaction was observed after 48 h. The percentage of cells stained positively was 95–100.

A substantial increase in expression can be seen after the application of CaEP to the Cav3.2 subunit. The use of EP resulted in a minor increase in subunit expression. The immunocytochemical analysis carried out in this study showed that for both tested cell lines, the use of CaEP resulted in a strong increase in the expression of both tested subunits, especially Cav3.2. So far, no detailed research in this direction on breast cancer has been conducted.

The analysis of the cell morphology showed changes after the use of EP and CaEP. The most characteristic observed changes were apoptotic bodies, typical of the phenomenon of apoptosis (visible in the form of multiple obstructed vesicles). After the application of electroporation and CaEP to the cells, the presence of fibrous material in the cytoplasm was observed. Dense packing of vacuoles in the cytoplasm was also present. Similar results were obtained by the research team of Starasenic B. et al. [34]. After applying CaEP, researchers observed changes in cell morphology and the presence of apoptotic bodies. Skołucka N. et al. demonstrated the presence of multiple vacuoles in cells of the Me45 (human epidermoid carcinoma) cell line after EP application [35]. CaEP is based on the cell-lethal intracellular calcium level, which is generated by the electropermeabilization of tumor tissue. The loss of calcium homeostasis that occurs with CaEP use can lead to cell death by necrosis, apoptosis, or autophagy [24].

The first experiment using CaEP in vitro was carried out in 2003 [36]. It has been shown that calcium ions enter the cells after electroporation in a buffer containing calcium ions, and the high extracellular concentration of Ca^2+^ during electroporation reduces their survival. CaEP was first described as a new anticancer therapy in 2012 [37]. Studies have also shown that CaEP is more effective in inducing cell death in cancer cells than in normal cells [12,38]. The reason for this may be the increased expression of calcium pump proteins in normal cells compared to neoplastic cells, which allows the former to remove excess calcium from the cytosol faster [34].

In the presented paper, the type of induced cell death was analyzed by flow cytometry using APC-annexin V (an apoptosis marker) and Sytox Green (a necrosis marker). Frandsen et al. compared the sensitivity of various human neoplastic and normal cell lines to electroporation with calcium ions. The results of the above experiment showed that electroporation with calcium ions induces rapid and selective necrosis of solid tumors with limited deleterious effects on the surrounding neoplastic tissue [38]. Other studies have indicated the mechanism of apoptotic cell death induced by calcium ion electroporation when tested at the in vitro level [12,13]. Staresinic et al. investigated the type of cell death by flow cytometry. The results showed that cells after CaEP were mostly necrotic or late-apoptotic. In the HUVEC (human endothelial) cell line, approximately 40% of the cells were in the late apoptotic phase, and 20% of the cells were necrotic. In the FaDu (human squamous carcinoma) cell line, 40% of the cells were late-apoptotic and about 10% necrotic. For the CHO (hamster ovary) cell line, the highest number of late-apoptotic cells was observed (60%). The B16F1 (mouse melanoma) cell line appeared to be the most sensitive to CaEP. As many as 70% of cells were in the late-apoptotic phase, and 10% of cells were necrotic. High efficiency of CaEP has been demonstrated in in vitro studies with the use of calcium chloride at concentrations of 1–5 mM in various types of tumors [34].

The results of flow cytometry and the TUNEL method presented in this paper are consistent with the above results. In the MCF-7/WT cell line, a large number of late apoptotic and necrotic cells were observed after CaEP application. The number of necrotic cells increased with the electric field strength parameters. In the MCF-7/DOX cell line, after the application of CaEP, the number of viable cells was higher than in the MCF-7/WT cell line. Most of the cells were in the early apoptosis phase. The number of late-apoptotic and necrotic cells was significantly lower for the same parameters in the MCF-7/DOX cell line.

After using the TUNEL method, it was observed that both electroporation and CaEP caused cell apoptosis. However, after EP, the percentage of positively stained nuclei was lower than with CaEP. In the MCF-7/WT cell line, the percentage of apoptotic cells was highest for the following parameters: 1200 V/cm + 5 mM (55%), 1400 V/cm + 1 mM (40%), and 1400 V/cm + 5 mM (70%). For the MCF-7/DOX line, the percentage of apoptotic cells was the highest for the same parameters and amounted to 1200 V/ cm + 5 mM (70%), 1400 V/cm + 1 mM (40%), and 1400 V/cm + 5 mM (50%).

Frandsen et al. tested the effect of CaEP on various types of tumors in vivo. Various types of cancer have been treated with CaEP: colorectal cancer (HT 29), adenocarcinoma of the mammary gland (MDA-MB-231), and bladder cancer (SW780). Post-treatment necrotic tissue size was estimated. CaEP induced tumor necrosis in all treated tumors, with a significantly higher fraction of necrosis in the two tumor types two days after treatment compared to the pre-treatment state (H69, MDA-MB-231) [12]. In lung cancer in vivo, complete necrosis was observed in 89% of tumors after CaEP [39]. In addition, a case report has been published on a patient with a systemic immune response and remission of disseminated malignant melanoma after ECT with bleomycin and CaEP [26,40]. Zielichowska et al. investigated the effect of CaEP on the murine fibrosarcoma cell line, Wehi-164, and normal cells of the rat skeletal muscle, L6. Wehi-164 (mouse fibrosarcoma) cells showed greater sensitivity to CaEP than L6 cells. The most significant antitumor effect—about 80% of apoptotic cells—was observed after the application of CaEP with the following parameters: 1500 V/cm, with the concentration of calcium chloride at 5 mM [12].

The effect of calcium ion-induced changes in combination with electroporation on the intracellular processes of mammary adenocarcinoma cells has not been thoroughly investigated. However, the results of studies on CaEP in the treatment of adenocarcinoma of the mammary gland published in the last decade are very promising [41].

The results of this study indicate that calcium ions introduced into cells by electroporation resulted in a good anti-tumor effect for both cell lines, especially for MCF-7/DOX. The best result was observed with CaEP. The survival rate of cells treated with CaEP was significantly lower compared to cells treated with EP alone. The best results were obtained with higher parameters of the electric field strength. The results obtained in this study can be the basis for designing CaEP protocols to be applied at the in vivo level.

The results presented in the paper may contribute to the development of new methods of treating mammary gland adenocarcinoma and become the basis for further research. Due to the low cost of calcium chloride, its availability, and its safety, CaEP therapy can be easily implemented in most hospitals. Calcium chloride is officially approved as a clinical treatment compound as an infusion fluid. In addition, the CaEP method is based in essence on the selectivity of action, so it does not cause side effects such as those of classic chemotherapy.

## 4. Materials and Methods

### 4.1. Cell Culturing

As a model for our experiments, two breast adenocarcinoma cell lines were used: wild type-MCF-7/WT and doxorubicin-resistant type-MCF-7/DOX. Both cell lines were acquired from the National Institute of Oncology in Gliwice. Cells were maintained in cell culture medium DMEM (Dulbeco’s Modified Eagle Medium, Sigma-Aldrich, Saint Louis, MO, USA) with 10% fetal bovine serum (FBS; Sigma-Aldrich) and supplemented with antibiotics (penicillin/streptomycin; Sigma-Aldrich) at 37 °C and 5% CO_2_. For the experiments, cells were removed by trypsinization (trypsin 0.25%, Sigma-Aldrich) and neutralized by DMEM.

### 4.2. Calcium Chloride Solution

The stock solution of calcium chloride (CaCl_2_, Sigma-Aldrich) was dissolved in PBS (Sigma–Aldrich) at a concentration of 100 mM. This stock was diluted in EP buffer or DMEM medium to the following concentrations: 0.25, 0.5, 1, and 5 mM.

### 4.3. Electroporation Protocol

EP and CaEP were performed using the ECM 830 square wave electroporation system (BTX Harvard Apparatus, Syngen Biotech, Wroclaw, Poland). After trypsinization and centrifugation, cells were suspended at a concentration of 4 × 10^6^/mL in 300 μL EP buffer or 300 μL EP buffer with CaCl_2_ and electroporated in 4 mm cuvettes (BTX Harvard Apparatus, purchased from Syngen Biotech, Wroclaw, Poland). The EP buffer (conductivity σ_0_ = 0.12 S/m, pH 7.4) consisted of 250 mM sucrose, 10 mM phosphate buffer KH_2_PO_4_/K_2_HPO_4_, and 1 mM MgCl_2_. The cells were exposed to 8 pulses of intensity 800–1400 V/cm (100 μs duration at 1 Hz repetition rate) in the presence of the following calcium concentrations (0.25–5 mM) in EP buffer or only in EP buffer. Control cells were not electroporated but only incubated with EP buffer. After pulsation, the cells were left for 10 min at 37 °C, centrifuged (5 min, 1500 rpm), and resuspended for further experiments in a cell culture medium.

### 4.4. Cells Viability: The Evaluation of Cell Survival Was Conducted by Three Independent Methods

#### 4.4.1. MTT Assay

Cell viability after experiments (EP, CaEP, and CaCl_2_) was determined by the MTT assay, which evaluates mitochondrial activity. Cells were seeded on 96-well plates (Nunc) at 250 × 10^3^ cells/ well and incubated for 24 and 48 h. Then, cells were washed with PBS (phosphate buffered saline, Sigma–Aldrich) solution, and 100 μL/well of MTT [3-(4,5-dimethylthiazol-2-yl)-2,5-diphenyl tetrazolium bromide] (Sigma–Aldrich) was added. Cells were incubated for 1.5 h at 37 °C. The formazan crystals were dissolved by adding 100 μL/well of isopropanol diluted in 0.1 M HCl. After thorough mixing, the absorbance value was measured at 570 nm by a multiwell microplate reader (Enspire^®^ Multiplate Reader, Perkin Elmer, Waltham, MA, USA). The obtained results were expressed as the percentage of viable cells relative to untreated control cells.

#### 4.4.2. SRB Assay

Cell viability after experiments (EP, CaEP, and CaCl_2_) was determined by the SRB assay, which evaluates the proteins in living cells. Sulforhodamine B is a dye that binds to the amino acids of cellular proteins. Cells were seeded on a 96-well plate (Nunc) at 250 × 10^3^ cells/well and incubated for 24 and 48 h. After incubation, cells resided at 50% trichloroacetic acid (30 min, 4 °C). After rinsing with water and drying, the cells were stained with sulforodamine B diluted in 1% acetic acid (30 min, 20 °C). Then, cells were washed with 1% acetic acid, dried out, and the protein-bound dye was dissolved in a 10 mM TRIS (Sigma–Aldrich) solution (30 min). The absorbance (492 nm) was measured by a multiwell microplate reader (Enspire^®^ Multiplate Reader, Perkin Elmer, Poland). The obtained results were expressed as the percentage of viable cells relative to untreated control cells.

#### 4.4.3. Flow Cytometry Study

In order to evaluate the efficiency of cell membrane permeabilization, cells were stained with a YO-PRO^TM^ -1-iodide fluorescent dye absorbed by the cell (YP-1, λ_em_ 488) and immediately electroporated. The following electric field strength parameters were used: 400, 600, 800, 1000, 1200, and 1400 V/cm. After electroporation, cells were centrifuged (2 min, 1800 rpm) and flow cytometer analysis was performed (Cube 6, Sysmex, Warszawa, Poland).

The analysis of the type of cell death was carried out by flow cytometry (FACS) with the use of SYTOX^TM^ Green (Sytox Green dead cell stain, Thermo Fisher Scientific, Waltham, MA, USA) and Annexin V (APC Annexin V Apoptosis Detection Kit with PL, BioLegend, San Diego, CA, USA). After experiments (CaEP), cells were diluted in culture medium, placed on a 6-well plate (1 × 10^4^/well), and incubated for 12 h. Then, the supernatant was removed to test tubes and centrifuged (6 min × 2000 rpm). The rest of the cells were trypsinized, washed with DPBS, and centrifuged (6 min, 2000 rpm). Then, 100 μL binding buffer, 5 μL of AnnexinV, and 1 μL of SYTOX Green were added. After mixing and incubating in the dark (15 min), 400 μL of DPBS was added to each sample. Flow cytometric analysis was performed using the CyFlow Cube 6 flow cytometer (Sysmex, Poland). Samples were excited with blue light (λ_em_ 525) for SYTOX Green and red light (λ_em_ 675) for Annexin V. The obtained results were expressed as the percentage of live, early-apoptotic, late-apoptotic, and necrotic cells.

### 4.5. Immunocytochemical Staining

The antibodies Cav3.1 (Sigma–Aldrich) and Cav3.2 (Santa Cruz Biotechnology, Dallas, TX, USA) were diluted in antibody diluent buffer (DAKO, 1:100). After EP, CaEP, and CaCl_2_ alone, the cells were seeded on slide covers and incubated for 24 and 48 h at 37 °C. Then, the samples were fixed in 4% formalin, and antibodies were applied for 24 h at 4 °C. The further protocol based on DAKO LSAB+ System-HRP (DAKO, Poland) began with washing in PBS with Triton for 3 × 5 min and adding secondary antibodies (biotinylated anti-mouse immunoglobulins in PBS) for 20 min. Again, the glasses were washed in PBS+ Triton, and streptavidin-HRP was applied for 20 min. Thereafter, the DAB+ chromogen diluted in DAB+ Substrate Buffer was added for 5 min (incubated in the darkness) and rinsed in distilled water for 10 min. Then, hematoxylin was used to colorize nuclei (1 min). In the following steps, dehydration was performed by increasing the concentration of ethanol and xylene and mounted in DPX Mounting Medium (Fluka, Germany). The upright microscope (Olympus BX51, Shinjuku, Tokio, Japan) was used to examine the immunocytochemical reaction. The intensity of staining was evaluated as (-) negative, (+) weak, (++) moderate, and (+++) strong.

### 4.6. Tunel Assay

In order to observe apoptosis, after experiments with EP and CaEP, cells were seeded on slide covers (8) and incubated for 24 h at 37 °C. Thereafter, the samples were fixed in 4% formalin and washed in PSB (3 × 5 min). The apoptosis detection was performed according to instructions using the Apoptosis Detection Kit (ApopTag, Peroxidase in Situ, EMD Millipore). The cells were permeabilized with proteinase K for 10 min, washed in PBS (3 × 5 min), and incubated with 2% H_2_O_2_ (5 min). After the incubation with TdT enzyme for 1 h at 37 °C, the reaction was stopped by Stop/Wash Buffer (10 min). Then, glasses were washed in PBS (3 × 5 min), and the anti-digoxigenin conjugate was applied for 30 min. The labeling was detected using the DAB solutions (5 min in the darkness), and the staining by hematoxylin (1 min) was performed. Finally, the cells were incubated in increasing concentrations of ethanol and xylene (3 × 5 min). Samples were closed in DPX mounting medium (Fluka). The effects of the experiment were observed with Olympus BX51 microscopy (Olympus, Shinjuku, Tokio, Japan).

### 4.7. Living Cell Tomographic Microscopy

Furthermore, to observe intracellular morphological changes, cells were visualized in the live cell tomographic holographic 3D microscope Nanolive (3D Cell Explorer, Nanolive, Labnatek, Warszawa, Polska). 3D Cell Explorer (STEVE, Nanolive) software allows for the visualization of organelles in living cells in detail. This microscope is also suitable for long-term imaging of living cells. After EP and CaEP, cells were suspended in DMEM, placed on coverslips, and incubated (24 h, 37 °C). Then, coverslips were placed on slides (Menzel-Glaser, Waltham, MA, USA) and subjected to imaging. Single cells or small cell aggregates were imaged.

### 4.8. Statistical Analysis

The experiments were performed in three replicates. The statistical analysis was performed using GraphPad Prism 7.03. Data were analyzed by two-way ANOVA with Dunnett’s multiple comparison tests, in which the test groups of cells treated with calcium ions were compared with the group of control cells not exposed to any action. Using the Sidak test, the studied groups of cells treated with CaEP were compared to control cells not exposed to any action and with a group of cells treated with EP alone.

## 5. Conclusions

The results presented in this work demonstrate that CaEP might be a potentially effective treatment for human breast adenocarcinoma cancer cells in vitro. Pre-incubation of both cell lines with a 5 mM CaCl_2_ solution assisted by electroporation resulted in the most effective anticancer activity. The observed effects of CaEP on human breast adenocarcinoma cells suggest that this method holds great potential for future therapeutic applications. By utilizing electroporation to facilitate the uptake of calcium chloride, we were able to achieve enhanced anticancer activity. This discovery lays the groundwork for the development of new treatment protocols to be explored in vivo and in clinical settings. However, it is important to note that additional research and safety validation will be required before this method can be implemented in humans. Nonetheless, the results obtained thus far demonstrate the potential of CaEP and provide a starting point for the development of future treatment protocols.

## Figures and Tables

**Table 1:** Evaluation of apoptosis using the TUNEL method in MCF-7/WT and MCF-7/DOX cells after EP and CaEP in both cell lines.

	MCF-7/WT	MCF-7/DOX
	positively stained nucleus (%)	
control cells	0	0
800 V/cm	10	10
800 V/cm + 0.25 mM Ca	20	8
800 V/cm + 0.5 mM Ca	15	12
800 V/cm + 1 mM Ca	20	15
800 V/cm + 5 mM Ca	30	20
1000 V/cm	15	7
1000 V/cm + 0.25 mM Ca	20	8
1000 V/cm + 0.5 mM Ca	25	10
1000 V/cm + 1 mM Ca	40	12
1000 V/cm + 5 mM Ca	30	28
1200 V/cm	20	12
1200 V/cm + 0.25 mM Ca	15	5
1200 V/cm + 0.5 mM Ca	20	10
1200 V/cm + 1 mM Ca	20	12
1200 V/cm + 5 mM Ca	55	70
1400 V/cm	30	20
1400 V/cm + 0.25 mM Ca	30	10
1400 V/cm + 0.5 mM Ca	25	20
1400 V/cm + 1 mM Ca	40	40
1400 V/cm + 5 mM Ca	70	50

**Table 2:** Intensity of the immunocytochemical reaction of the Cav3.1 subunit of the T-type calcium channel with the percentage of cells stained positively after the application of EP and CaEP.

	[%] of Positively Stained Cells and Intensity of the Immunocytochemical Reaction			
Cav3.1	MCF-7/WT		MCF-7/DOX	
	24 h	48 h	24 h	48 h
control cells	+/− 50	+/− 70	+/− 50	+/− 80
800 V/cm	+ 50	+/− 70	+/− 80	+/++ 90
800 V/cm + 0.25 mM Ca	+/++ 50	+/++ 60	+ 70	++ 90
800 V/cm + 0.5 mM Ca	+/++ 70	+/++ 70	+/++ 70	++ 90
800 V/cm + 1 mM Ca	++/+++ 90	++ 90	+/++ 90	++/+++ 95
800 V/cm + 5 mM Ca	++/+++ 95	++/+++ 98	++/+++ 98	++/+++ 98
1000 V/cm	+/++ 80	+ 80	+ 80	++ 80
1000 V/cm + 0.25 mM Ca	+/++ 90	+/++ 90	++ 90	++ 90
1000 V/cm + 0.5 mM Ca	++ 90	++ 95	++ 95	++ 90
1000 V/cm + 1 mM Ca	+/++ 90	++/+++ 98	++ 95	++/+++ 95
1000 V/cm + 5 mM Ca	++/+++ 95	+++ 100	++/+++ 98	++/+++ 98
1200 V/cm	++ 70	+/++ 90	+/++ 80	++/+++ 90
1200 V/cm + 0.25 mM Ca	++ 90	++ 95	+/++ 90	+/++ 90
1200 V/cm + 0.5 mM Ca	++ 95	++/+++ 95	++ 95	++ 95
1200 V/cm + 1 mM Ca	+++ 100	+++ 98	++ 98	++ 95
1200 V/cm + 5 mM Ca	+++ 100	+++ 98	++/+++ 100	++/+++ 100
1400 V/cm	++ 90	++ 95	++ 90	++ 90
1400 V/cm + 0.25 mM Ca	++ 95	+/++ 90	++ 90	++ 95
1400 V/cm + 0.5 mM Ca	++ 95	++ 95	++/+++ 95	++/+++ 98
1400 V/cm + 1 mM Ca	++/+++ 98	+++ 98	++/+++ 98	++/+++ 100
1400 V/cm + 5 mM Ca	+++ 100	+++ 100	+++ 100	+++ 100

The intensity of staining: (−) negative, (+/−) trace, (+) weak, (++) moderate, (+++) strong.

**Table 3:** Intensity of the immunocytochemical reaction of the CaV 3.2. subunit of the T-type calcium channel with the percentage of cells stained positively after the application of EP and CaEP.

	Percentage of Positively Stained Cells and Intensity of the Immunocytochemical Reaction			
Cav3.2	MCF-7/WT		MCF-7/DOX	
	24 h	48 h	24 h	48 h
control cells	+/− 50	+/− 30	+/− 50	+/− 90
800 V/cm	+ 70	+ 50	+/− 90	0
800 V/cm + 0.25 mM Ca	+/++ 100	++ 95	++ 100	+/++ 98
800 V/cm + 0.5 mM Ca	+ 90	++ 98	++/+++ 100	++ 98
800 V/cm + 1 mM Ca	+/++ 70	+/++ 95	++ 100	++ 100
800 V/cm + 5 mM Ca	++ 95	++ 100	++ 100	++ 100
1000 V/cm	+ 70	+/++ 70	+ 98	0
1000 V/cm + 0.25 mM Ca	+/++ 90	+/++ 95	+/++ 90	+ 70
1000 V/cm + 0.5 mM Ca	++ 100	++ 100	+ 90	++ 100
1000 V/cm + 1 mM Ca	+/++ 90	++ 98	++/+++ 100	+ 90
1000 V/cm + 5 mM Ca	++ 100	++ 100	++ 100	++/+++ 100
1200 V/cm	+/++ 90	++ 90	+/++ 98	+ 90
1200 V/cm + 0.25 mM Ca	+/++ 70	++ 95	++ 100	++ 98
1200 V/cm + 0.5 mM Ca	++ 90	+/++ 95	++/+++ 100	++ 90
1200 V/cm + 1 mM Ca	++ 90	++/+++ 90	++/+++ 100	++/+++ 100
1200 V/cm + 5 mM Ca	++/+++ 95	+++ 100	++/+++ 100	++/+++ 100
1400 V/cm	+/++ 98	++ 98	++ 98	+/++ 98
1400 V/cm + 0.25 mM Ca	++ 100	+++ 100	++/+++ 100	++ 90
1400 V/cm + 0.5 mM Ca	++ 90	++/+++ 100	+++ 100	+++ 98
1400 V/cm + 1 mM Ca	++ 90	++ 98	++ 100	++/+++ 100
1400 V/cm + 5 mM Ca	++ 100	+++ 100	++/+++ 98	++/+++ 100

The intensity of staining: (−) negative, (+/−) trace, (+) weak, (++) moderate, (+++) strong.

## Data Availability

The data presented in this study are available on request from the corresponding authors. The data are not publicly available due to the consent provided by participants for the use of confidential data.

## Supplementary Materials

The following supporting information can be downloaded at: https://www.mdpi.com/article/10.3390/ph16060809/s1, Figure S1: Expression of the Cav3.1 subunit after 24-h incubation in the MCF-7/WT cell line; Figure S2: Expression of the Cav3.1 subunit after 48-h incubation in the MCF-7/WT cell line; Figure S3: Expression of the Cav3.1 subunit after 24-h incubation in the MCF-7/DOX cell line, Figure S4: Expression of the Cav3.1 subunit after 48-h incubation in the MCF-7/DOX cell line; Figure S5: Expression of the Cav3.2 subunit after 24-h incubation in the MCF-7/WT cell line; Figure S6: Expression of the Cav3.2 subunit after 48-h incubation in the MCF-7/WT cell line; Figure S7: Expression of the Cav3.2 subunit after 24-h incubation in the MCF-7/DOX cell line, Figure S8: Expression of the Cav3.2 subunit after 48-h incubation in the MCF-7/DOX cell line.
