# Supplementary material for: Evaluation of the Anticancer Activity of Calcium Ions Introduced into Human Breast Adenocarcinoma Cells MCF-7/WT and MCF-7/DOX by Electroporation
Source: Pharmaceuticals (Basel). 2023 May 30;16(6):809. doi: 10.3390/ph16060809 (PMC10304440; doi:10.3390/ph16060809)
Supplement: Supplementary file 1 [file pharmaceuticals-16-00809-s001.zip › pharmaceuticals-2358017-supplementary.pdf]

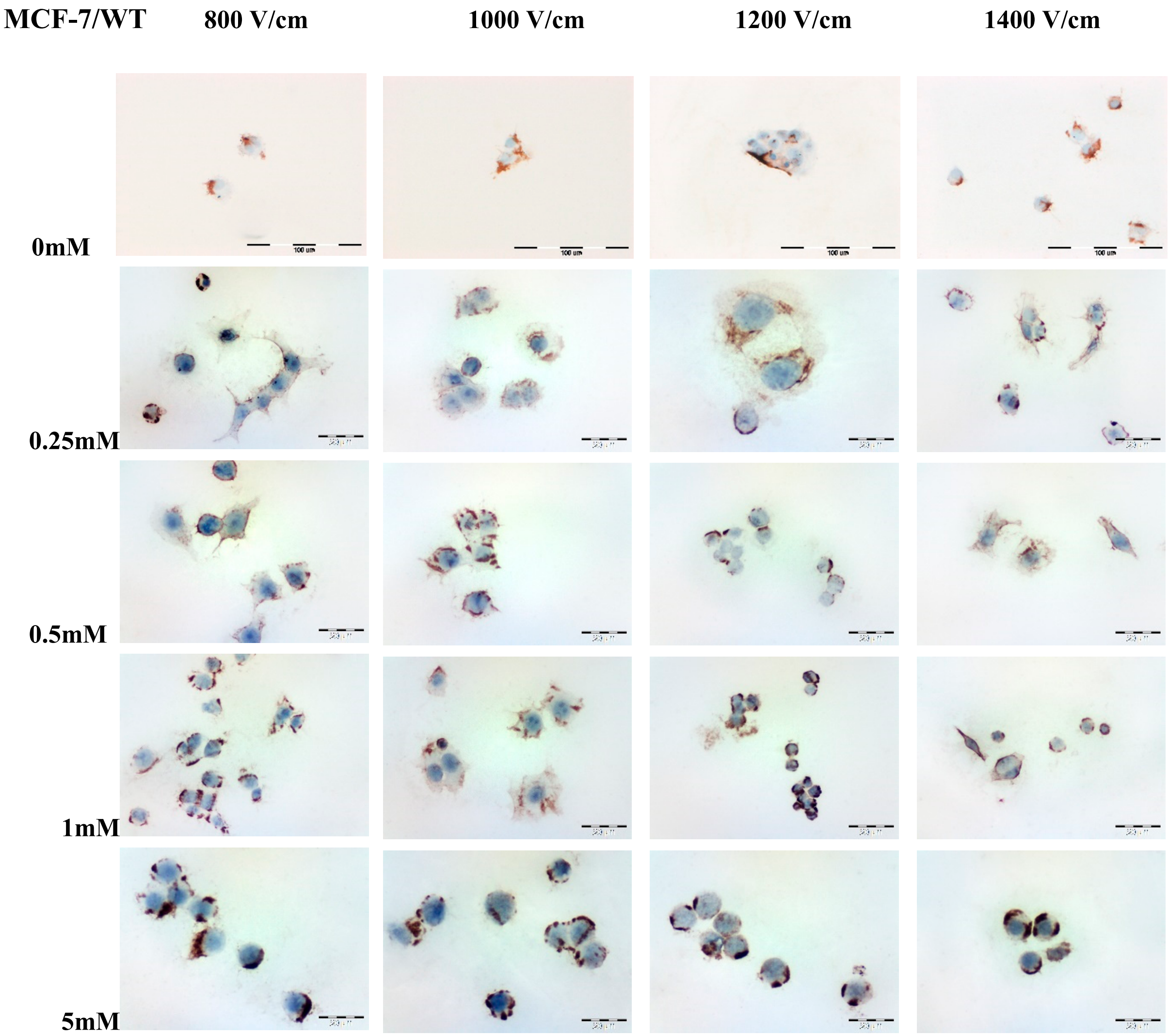

Figure S1. Expression of the Cav3.1 subunit after 24-hour incubation in MCF-7/WT cell line.

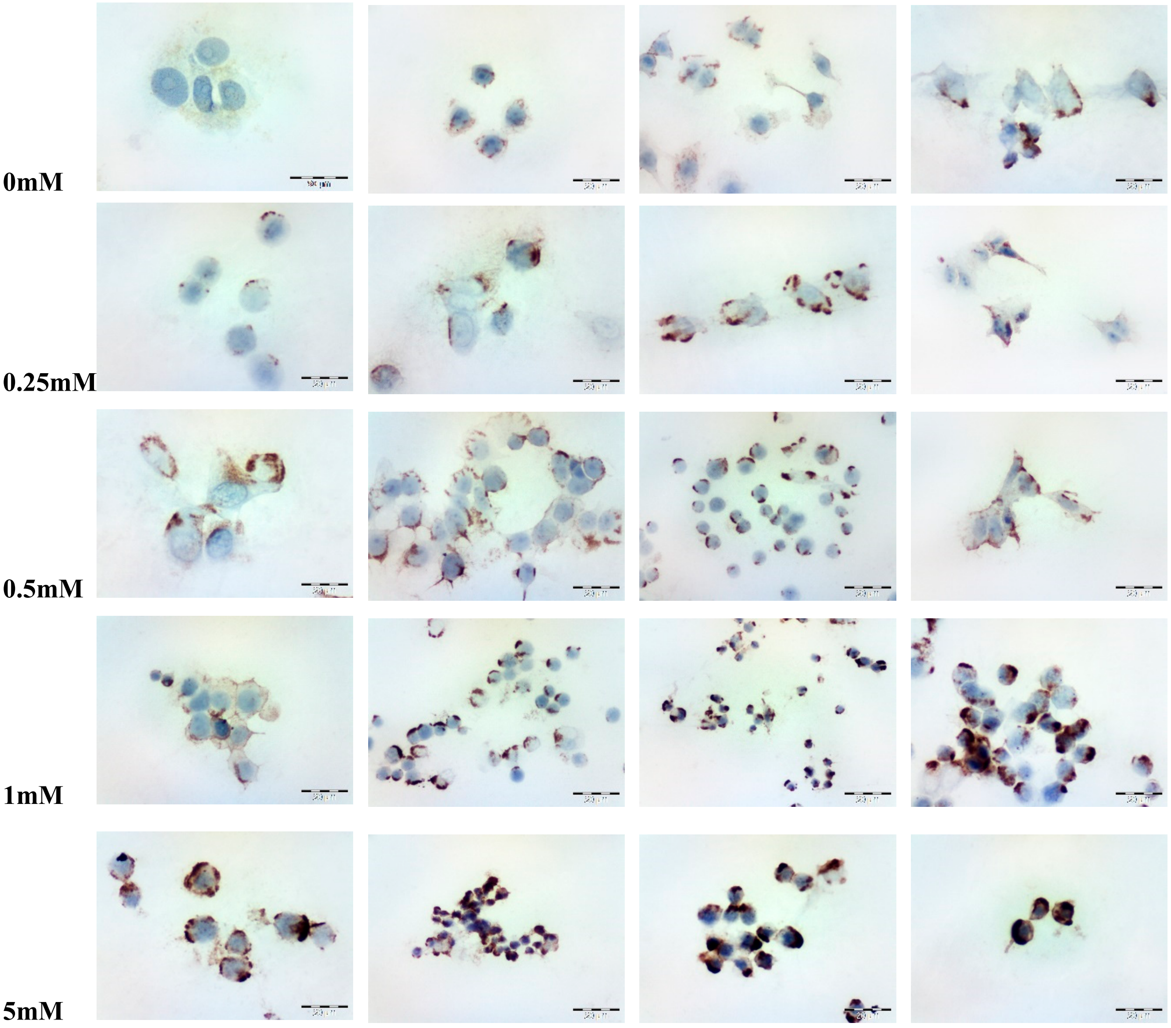

Figure S2. Expression of the Cav3.1 subunit after 48-hour incubation in MCF-7/WT cell line.

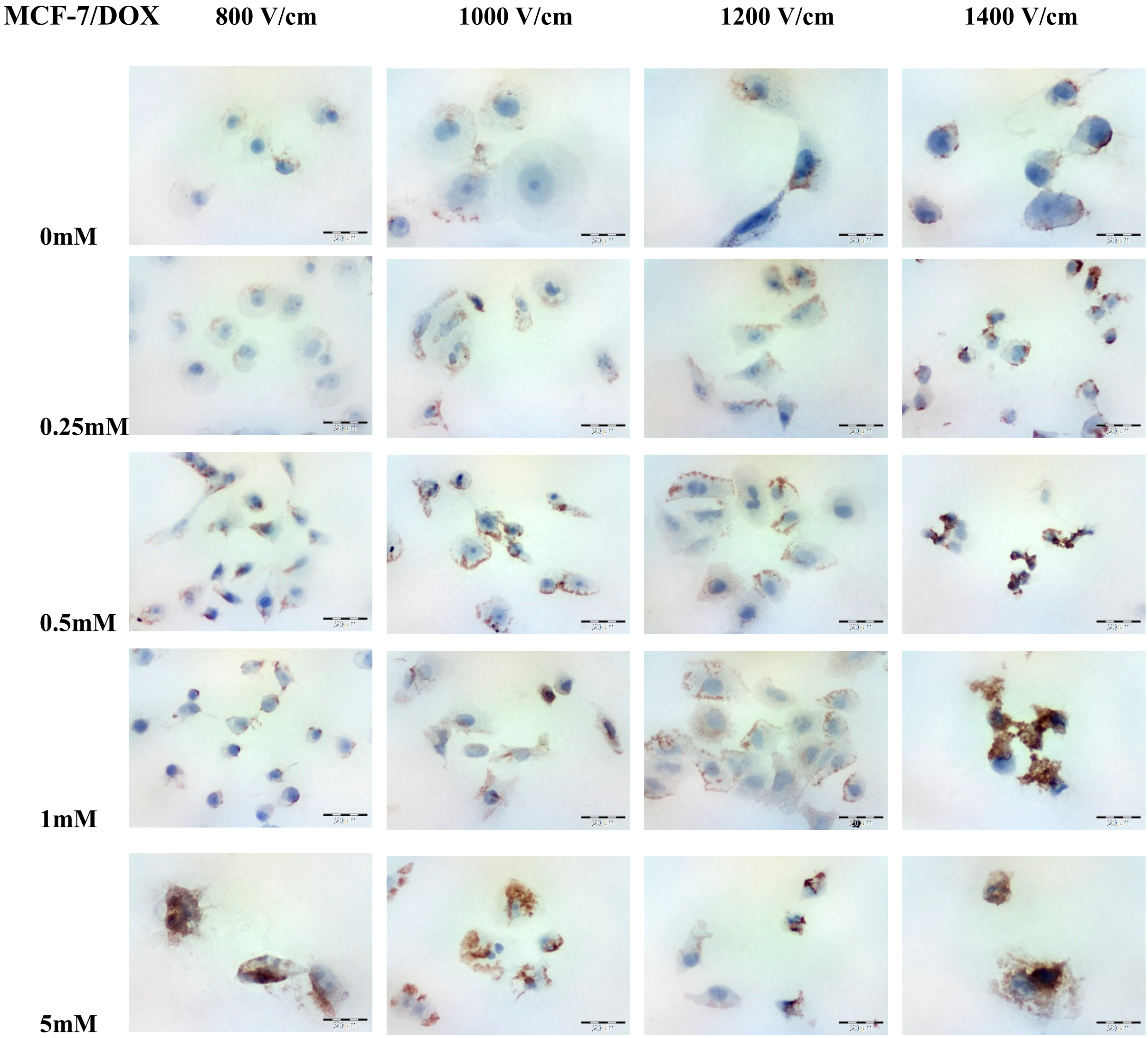

Figure S3. Expression of the Cav3.1 subunit after 24-hour incubation in MCF-7/DOX cell line.

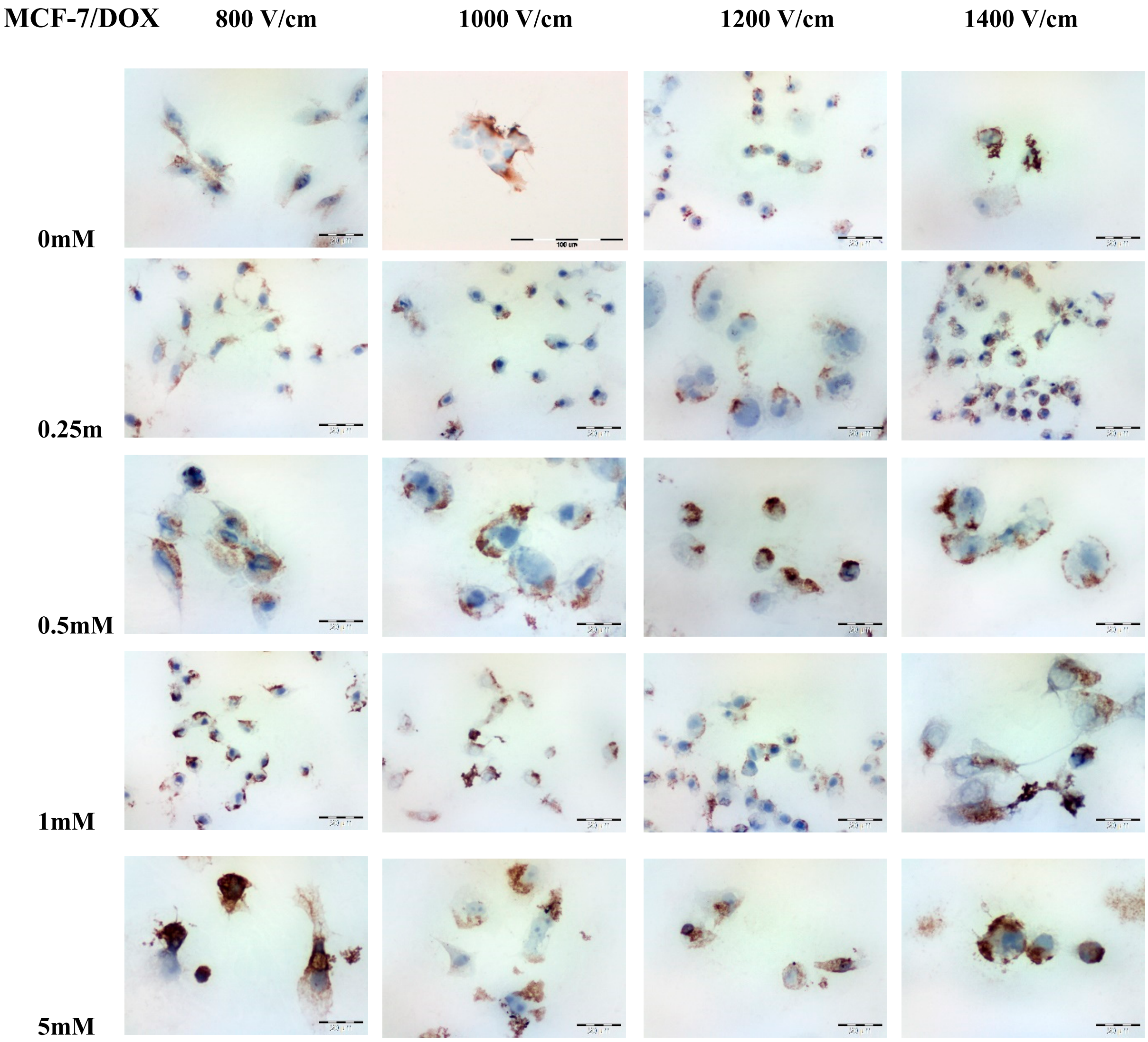

Figure S4. Expression of the Cav3.1 subunit after 48-hour incubation in MCF-7/DOX cell line.

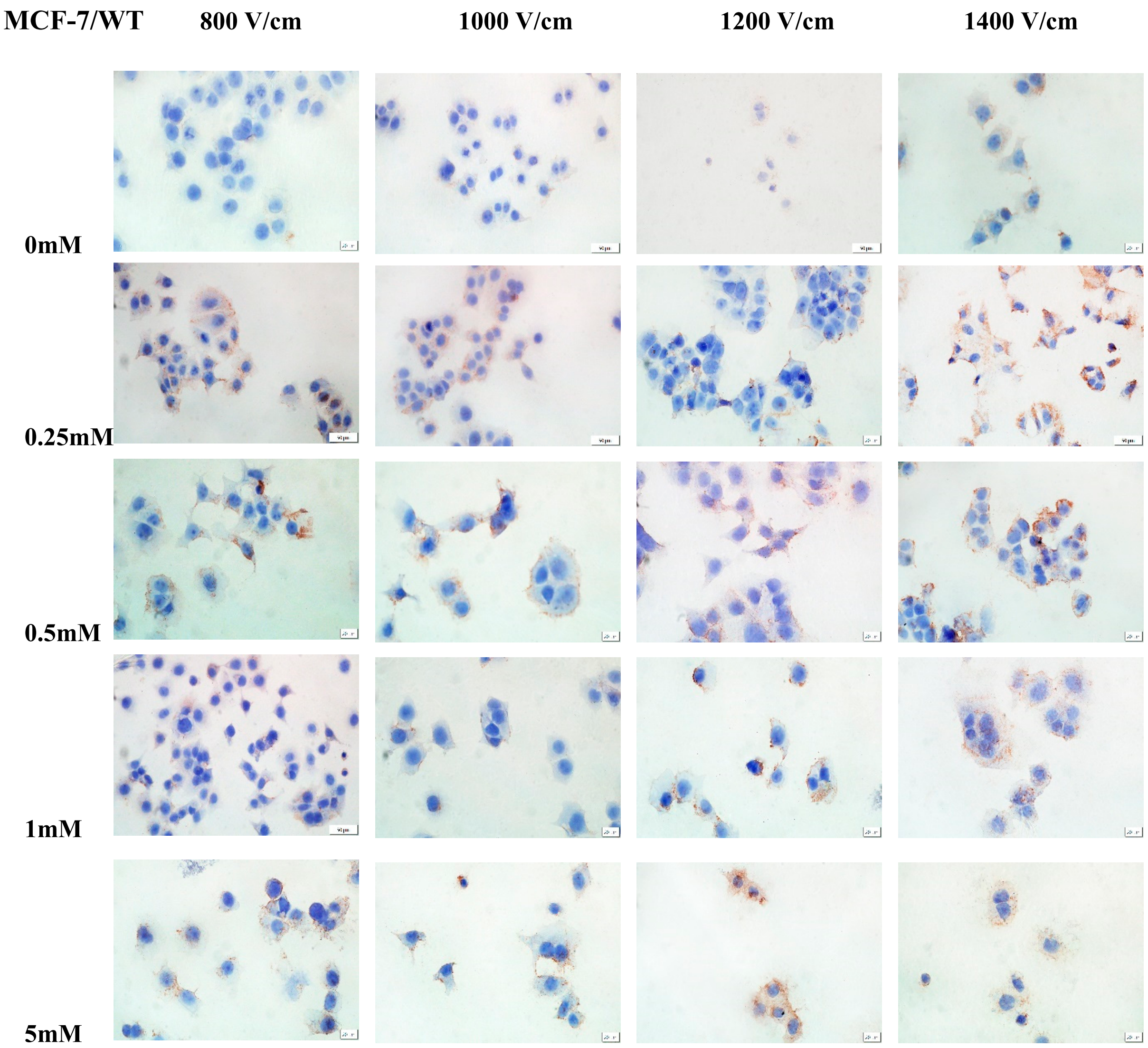

Figure S5. Expression of the Cav3.2 subunit after 24-hour incubation in MCF-7/WT cell line.

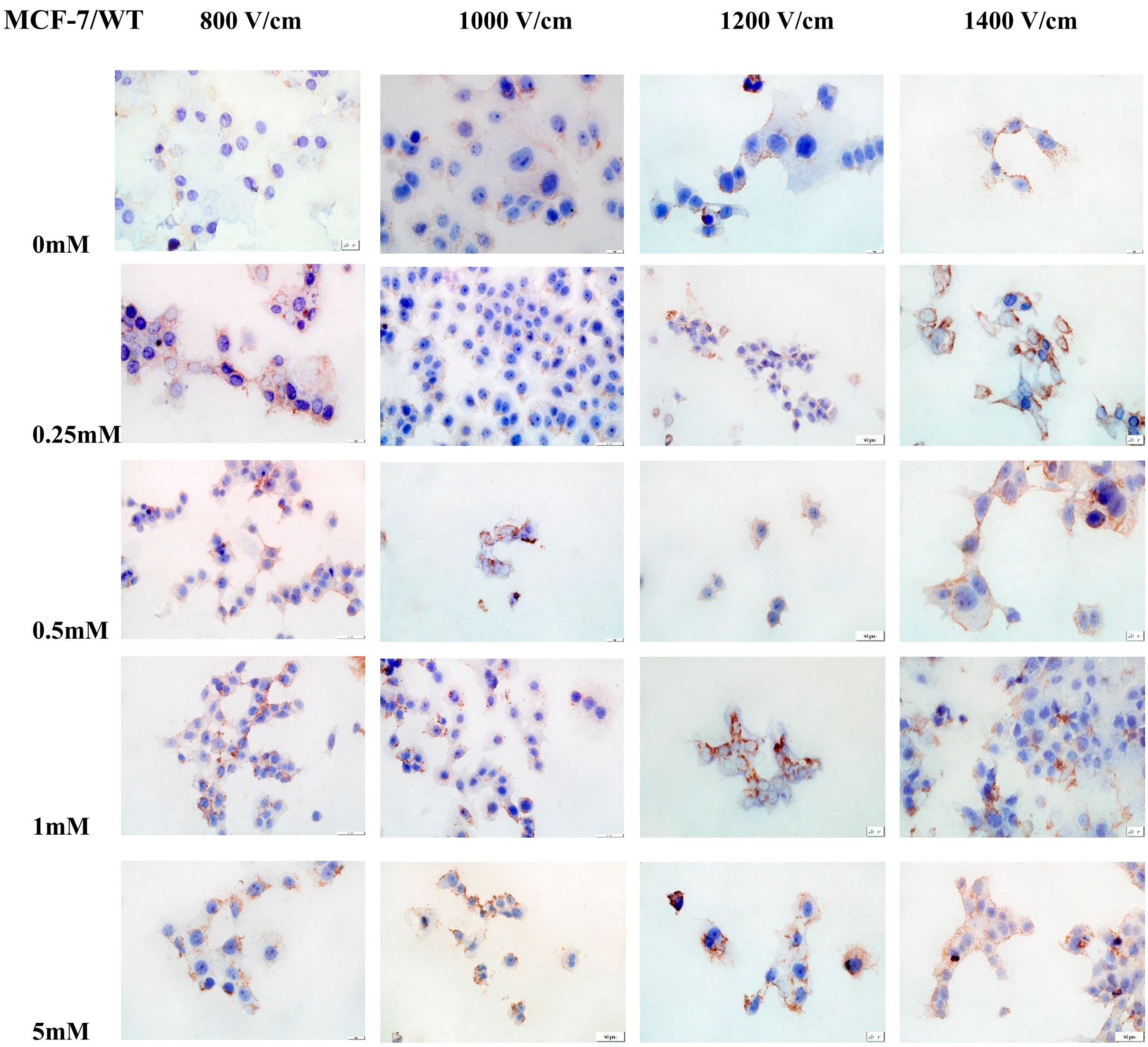

Figure S6. Expression of the Cav3.2 subunit after 48-hour incubation in MCF-7/WT cell line.

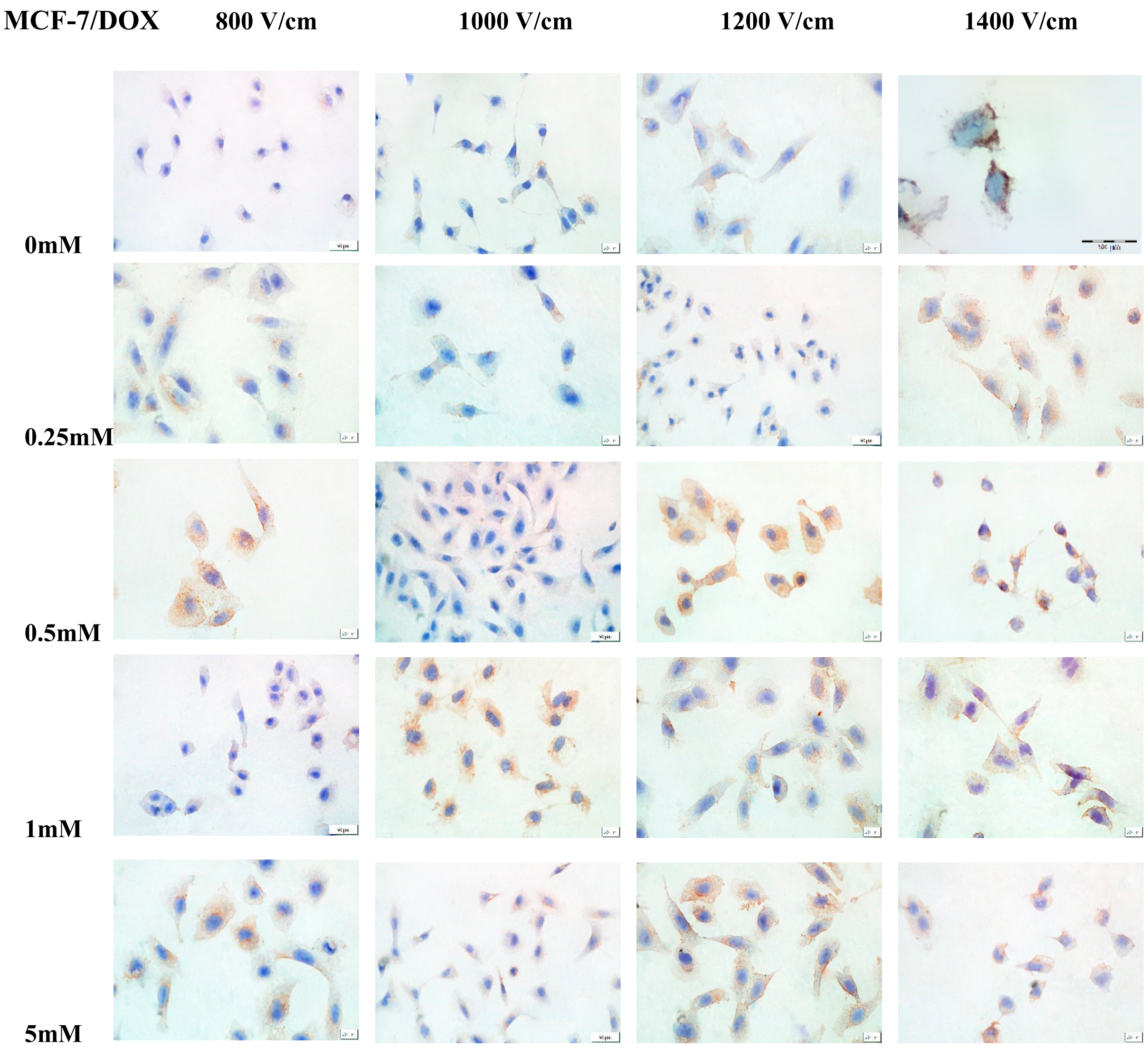

Figure S7. Expression of the Cav3.2 subunit after 24-hour incubation in MCF-7/DOX cell line.

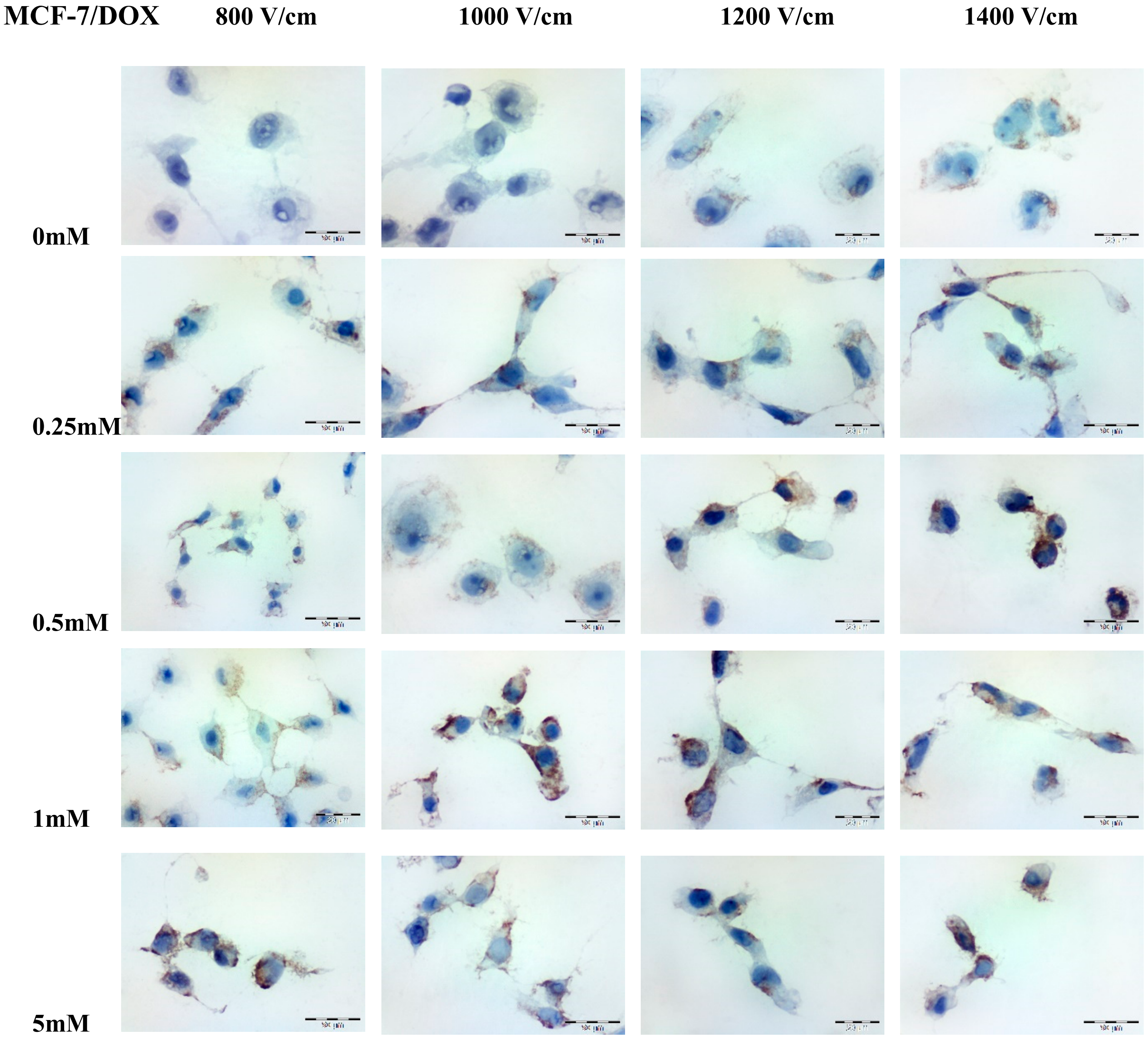

Figure S8. Expression of the Cav3.2 subunit after 48-hour incubation in MCF-7/DOX cell line.
